# Dual functional nisin-multi-walled carbon nanotubes coated filters for bacterial capture and inactivation

**DOI:** 10.1186/s13036-015-0018-8

**Published:** 2015-10-24

**Authors:** Xiuli Dong, Liju Yang

**Affiliations:** Biomanufacturing Research Institute and Technology Enterprise (BRITE) and Department of Pharmaceutical Sciences, North Carolina Central University, Durham, NC 27707 USA

**Keywords:** Bacterial pathogens, Filters, Carbon nanotubes, Nisin, Capture, Inhibition

## Abstract

**Background:**

Removal of pathogens from water is one way to prevent waterborne illness. In this paper, we developed dual functional carbon nanotube (CNT) modified filters for bacterial capture and inactivation, utilizing multi-walled CNTs (MWCNTs) to coat on commercially available filters and making use of the exceptional adsorption property of CNTs to adsorb a natural antimicrobial peptide-nisin on it. Two types of MWCNTs with different outer layer diameters were used (MWCNTs1: <8 nm in diameter; MWCNTs2: 10–20 nm in diameter).

**Results:**

The thickness of MWCNT layers, surface morphology, and surface hydrophobicity of both types of MWCNT coated filters were characterized. The MWCNT coating on filters significantly increased the surface hydrophobicity. The absorption of nisin and the capture of bacterial pathogens were correlated with increased surface hydrophobicity. The MWCNTs1 and MWCNTs2 filters with 1.5 mg MWCNTs loading captured 2.44 and 3.88 log of cells, respectively, from aqueous solutions containing a total of ~10^6^ CFU/mL cells. Nisin deposit at the amount of 0.5 mg on the surfaces of MWCNT filters significantly reduced the viability of captured *B. anthracis* cells by 95.71–97.19 %, and inhibited the metabolic activities of the captured cells by approximately 98.3 %.

**Conclusions:**

The results demonstrated that the MWCNT-nisin filters achieved dual functions in bacterial pathogen capture and inhibition in one single filtration step, which is potentially applicable in removing undesired microorganisms from water sources and inhibiting captured Gram positive bacteria activities.

## Background

Pathogenic microorganism contamination in water can cause waterborne diseases in human beings and animals. Common waterborne pathogenic microorganisms include various bacteria, viruses, and protozoa. According to World Health Organization, over 3.4 million people die from water-related diseases each year [1]. However, effective control of pathogenic microorganism contamination in water can be a significant contribution to ensure water quality and to prevent waterborne infectious diseases throughout the world. Currently, various methods are developed or under investigation for the elimination, inactivation, or removal of pathogens from water. One commonly used category of methods relies on the use of various antimicrobial chemical treatments, such as adding gaseous chlorine (Cl_2_), liquid sodium hypochlorite, chloramine, chlorine dioxide, chloramines, hydrogen peroxide, or bromine into water. These chemicals are effective in inactivating pathogens, but at the same time pose risks in environmental safety and public health. For instance, when sodium hypochlorite is used at high concentrations, it causes mucous membrane irritation, infra-clinical damage on intact skin [2], deterioration in organoleptic quality, an unpleasant odor, residual chlorine, and production of byproducts including the carcinogenic substance trihalomethane [3, 4]. In some cases, direct contact with high concentrations of antimicrobial agents may cause extensive damage to biological systems (like human tissues) [5]. The use of lower concentrations of the antimicrobial agents may minimize such risks, but in many cases, low concentrations definitely compromise the antimicrobial effect [6]. Another class of widely accepted methods is filtration-based methods, which is recognized as an effective way to remove microorganisms from contaminated water without posing side effects on environmental safety or health issues. Besides the use of commercially available membrane filters, recent studies have demonstrated the promising application of nanomaterial modified filters for this purpose. Carbon nanotubes (CNTs) have been proven as good candidates for modifying filters for effective removal of pathogens. CNTs are tubular cylinders of carbon atoms that possess unique mechanical, electrical, optical, thermal, and chemical properties. They have been considered as a novel and promising class of nanomaterials for applications such as new structural and functional materials, electrical circuitry, energy storage, drug delivery, biosensor devices, and many other devices of the future generation [7]. CNTs have also been exploited for a wide range of biological applications [8, 9]. Noticeably, CNTs modified filters/membranes have been reported, and show high permeability towards water and gases [10, 11], as well as the ability to remove bacterial and virus particles from water samples due to CNTs’ exceptional adsorption properties [12, 13]. In addition, CNTs modified filters/membranes also exhibited perceivable antimicrobial effects on the pathogens trapped on them, but not to a substantial level [14]. While exploring their promising applications, CNTs’ toxicity has been a concern when practical applications are considered. Most of the toxicity studies have been conducted using human cells [15–17] and animal models [18, 19], and the results have shown their adverse effects [18]. However, in many cases, functionalized CNTs showed promising biocompatibility [20].

In this paper, we developed dual functional CNT modified filters for bacterial capture and inactivation, utilizing multi-walled CNTs (MWCNTs) to coat on commercially available filters and making use of the exceptional adsorption property of CNTs to adsorb nisin, a natural antimicrobial peptide, on it. MWCNTs possess hollow cores with a large surface area to volume ratio, and this characteristic is well suited for physical adsorption of antimicrobial reagent molecules. Nisin is a non-toxic polycyclic peptide produced by certain strains of food-grade lactic acid bacterium during fermentation. It has been approved and used as a natural, toxicologically safe food preservative [21] in more than 50 countries including the US, the European Union, Brazil, and China. Nisin exhibits a broad spectrum of inhibitory activity against Gram-positive bacteria with high efficiency at nanomolar levels [22, 23]. Due to its amphiphilic nature, nisin is able to be adsorbed on both hydrophobic and hydrophilic surfaces [24], and can also be covalently linked to surface substrate or incorporated into polymer films. In fact, it has been used in food packaging films or other films for reducing foodborne pathogens. For example, several studies have shown that nisin incorporated into polyethylene films or polylactic acid polymer films significantly inhibited/inactivated foodborne bacterial pathogens [25] [26]. Our group has previously reported a study on a MWCNTs sheet on poly(methyl methacrylate) (PMMA) film for adsorbing nisin to provide anti-biofilm function [27]. In this study, the combination of nisin with CNTs-coated filters is expected to provide dual functions of highly efficient bacterial capture and highly effective bacterial inactivation in one simple filtration step. We used two different sized MWCNTs to coat on commercial Isopore polycarbonate hydrophilic membrane filters (25 mm in diameter and 5 μm pore size, EMD Millipore) for adsorption of nisin, characterized the surface properties of the resulting filters, and investigated the efficiencies of the resulting functional filters in capture and inactivation of bacterial pathogens from water using Gram-positive *Bacillus anthracis* cells as a testing microorganism.

## Results and Discussion

### Surface properties of MWCNTs coated filters

**Fig. 1:**
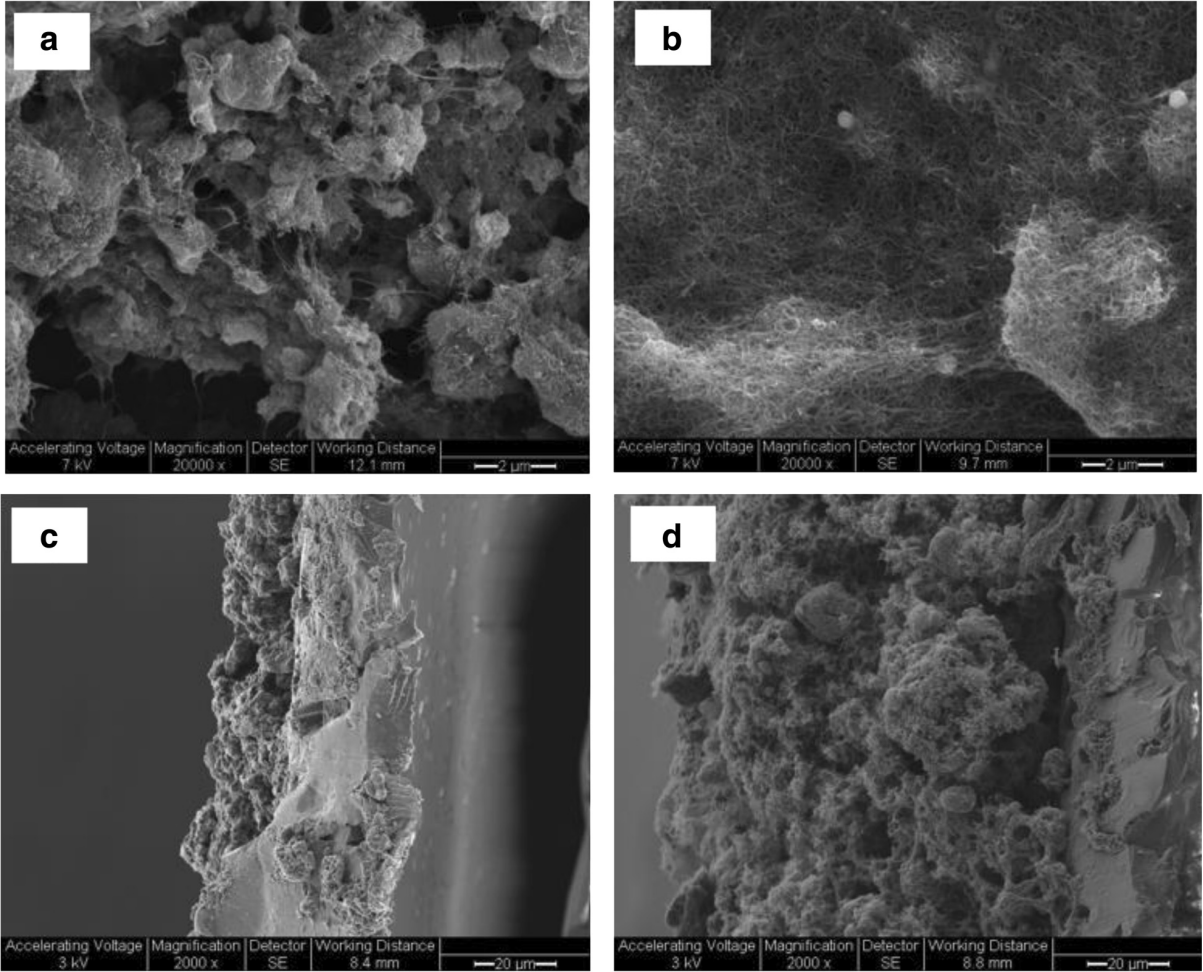
SEM images of MWCNTs coated filters, (**a**) and (**b**) the top view of MWCNTs on filters coated with MWCNTs1 and MWCNTs2, and (**c**) and (**d**) the cross section of filters coated with 1.2 mg MWCNTs 1 and MWCNTs 2

The use of microporous TMTP filters for coating with MWCNTs allowed for high water fluxes at low operating pressures while providing a sturdy base under the thin MWCNTs coating layer. We first used SEM imaging to examine the MWCNTs coatings on the TMTP filters. The SEM images indicated that 1.2 mg MWCNTs1 or MWCNTs2 were able to form a fairly sturdy layer on a TMTP membrane (4.91 cm^2^), whereas smaller loadings formed thinner layers. Sometimes, the washing steps with ethanol and deionized water (DI H_2_O) caused uneven distribution of both MWCNTs1 and MWCNTs2 on the filters, so precautions must be taken to avoid severe aggregations of MWCNT during the coating and washing steps. Figure 1a and b show the top view of MWCNTs1- and MWCNTs2- coated filters, showing that the morphology of the two types of MWCNTs on the filters differed, mostly likely due to the differences in nanotube sizes and their properties. While MWCNTs1 coating on the filters presented lumpy textures and more aggregates (Fig. 1a), the MWCNTs2 coating showed puffier textures, even distribution with individual tubes clearly seen, and fewer aggregations (Fig. 1b). The morphological difference in the coatings of MWCNTs1 and MWCNTs2 are most likely due to their size-associated properties, as it is commonly observed that smaller nanotubes are more likely to bundle due to their relatively large surface area and strong van der Waals interactions. The smaller nanotubes also have poorer dispersibility [28, 29], which likely made the coating more difficult and resulted in coated filters on which the distribution of the nanotubes was less homogeneous.

Next, the thicknesses of MWCNT layers was examined by SEM imaging the cross-sections of the coated filters. Figure 1c and d show the cross sections of the filters coated with 1.2 mg of MWCNTs1 and MWCNTs2. The thickness of MWCNTs1 layer and MWCNTs2 layer were 6.26 μm and 85.01 μm, respectively. With a lower loading of 0.6 mg MWCNTs, the thickness of MWCNTs1 layer and MWCNTs2 layer was also examined in the same way and found to be 3.35 μm and 30.11 μm, respectively. At both loading levels, the thickness of the MWCNTs2 layer was significantly higher (*P* < 0.05) than that of MWCNTs1 layer by approximately 10 times, which reasonably reflected the difference in the morphology/texture and the outer diameters between MWCNTs1 and MWCNTs2 as they were deposited on the filters. Also as expected, the thickness of MWCNT layers significantly increased (*P* < 0.05) with increasing loading amounts of MWCNTs from 0.6 to 1.2 mg in both cases, which is consistent with other reported CNTs coatings on various filters/membranes/films [30]. However, it is noted that increased thickness of the MWCNT layer contributed to narrower pores and higher surface area, which might decrease the water permeability according to the classical Kozeny-Carman equation for flow through porous filters [31, 32].

**Fig. 2:**
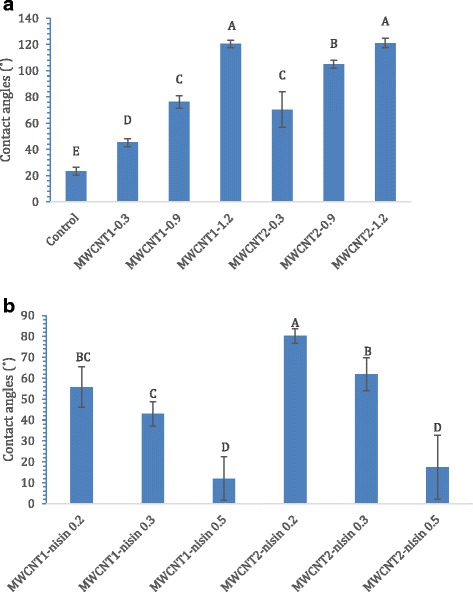
Surface contact angles of different filters, including (**a**) filters with 0.3, 0.9, and 1.2 mg MWCNT loadings; (**b**) filters with 1.2 mg MWCNT loading and 0.2, 0.3, or 0.5 mg nisin loading. Different letters above the bars indicate significant differences (*P* < 0.05). Different letters on the columns indicate a significant difference; the same letters on the columns indicate no significant difference

We then examined the surface hydrophobicity of the MWCNTs coated filters by measuring the contact angles of DI H_2_O droplets on the surfaces and the alterations of contact angles after nisin absorption on them, since the surface hydrophobicity is an important factor that affects the interactions between the surfaces and functional molecules. Figure 2a shows the surface contact angles of MWCNTs filters with various MWCNTs loadings. The TMTP membrane without MWCNTs loading was hydrophilic with the surface contact angle 23.4°. The contact angle increased to 45.23°, 76.10°, and 120.43° with 0.3, 0.9, and 1.2 mg MWCNTs1 loading, and similarly, the contact angle increased to 70.30°, 104.83°, and 121.03° with 0.3, 0.9, and 1.2 mg MWCNTs2 loading. The membrane surface exhibited significantly increased hydrophobicity after coating with MWCNTs (*P* < 0.05), as the surface contact angle was increased by 93.30 and 200.43 % with the loading of 0.3 mg MWCNTs1 and MWCNTs2, respectively. The realization of artificial hydrophobic surfaces is known to rely on two main features: the surface material chemical composition and its morphological structure [33, 34]. In this case, both types of MWCNT layers increased the surface hydrophobicity due to CNTs’ hydrophobic nature. These observations were in agreement with those in our previous study, in which both MWCNT forests on silicon wafers and MWCNT sheets on poly(methyl methacrylate) films significantly increased surface hydrophobicity (*P* < 0.05) [35]. It is also noted that the coating with MWCNTs1 and MWCNTs2 resulted in different surface hydrophobicity in certain situations, as the contact angles of MWCNTs2 surfaces were significantly higher (*P* < 0.05) than those of MWCNTs1 surfaces with 0.3 or 0.9 mg loading. However, at 1.2 mg MWCNTs loading, both types of MWCNTs coated filters showed similar contact angle values at which both surfaces exhibited very high hydrophobicity and no statistically significant difference (*P* > 0.05).

MWCNTs coated surfaces provided an ideal platform for nisin adsorption [27]. Interestingly, nisin absorption on MWCNTs decreased surface hydrophobicity of the filters, and the more nisin absorption, the lower the hydrophobicity was (Fig. 2b). With 0.2 mg nisin absorption, the surface contact angles decreased 53.70 % and 33.74 % on MWCNTs1 and MWCNTs2 filters, respectively, compared with the counterparts of MWCNTs filters without nisin. On the MWCNTs1 filter, the contact angle significantly decreased (*P* < 0.05) from 55.77° to 12.03° when 0.2 to 0.5 mg nisin was adsorbed. A similar trend was observed on MWCNTs2 filters, showing that the contact angles significantly decreased (*P* < 0.05) from 80.2° to 17.37° when nisin deposit increased from 0.2 to 0.5 mg. The characteristics of nisin’s molecular structure contributed to the surface property change. A nisin molecule contains 34 amino acid residues. The majority of the residues in the N-terminal are hydrophobic except a single charged residue Lys 12, whereas the C-terminal contains most charged and hydrophilic residues [36]. Upon absorption on MWCNTs’ surfaces, nisin molecules were mostly oriented with their hydrophobic side facing to the hydrophobic substrate and their hydrophilic side facing to the outer layers of the adsorbed peptide, which is in contrast to the observation on the hydrophilic substrates [37]. Such molecular orientation contributed to the high hydrophilic property (the lower contact angles) on the MWCNTs-nisin surfaces. Between MWCNTs1 and MWCNTs2 coated filters, it seems that more nisin was adsorbed on MWCNTs1 surfaces than on MWCNTs2 surfaces at low levels of nisin loading (0.2 or 0.3 mg), as the contact angles of MWCNTs1-nisin filters were significantly lower (*P* < 0.05) than those of MWCNTs2-nisin filters. This is understandable since MWCNTs1 had a much larger surface area than MWCNTs2 did; however, the contact angles of the two types of MWCNTs-nisin filters did not show a significant difference (P > 0.05) with 0.5 mg nisin loading, possibly because nisin absorption reached a saturation level on both MWCNTs surfaces.

### Efficiency of bacterial capture

**Fig. 3:**
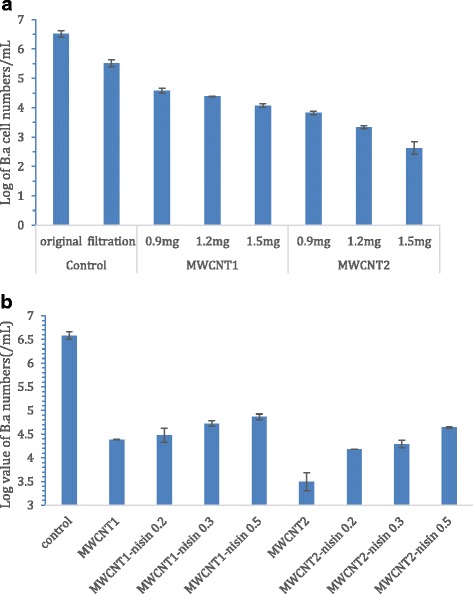
Log values of the living cells in the filtrates through the filters, including (**a**) the filters with 0, 0.9, 1.2, or 1.5 mg MWCNTs loading; (**b**) the filters with 1.2 mg MWCNT loading and 0, 0.2, 0.3, or 0.5 mg nisin loading

To evaluate the efficiency of the MWCNTs coated filters and nisin-MWCNTs coated filters for capturing bacteria from water, *B. anthracis* cells were used at the concentration of 3.3 × 10^6^ CFU/mL for testing. Figure 3a shows the log reductions in viable cell numbers in the filtrates after filtration with different MWCNTs coated filters. The TMTP membranes without MWCNTs and nisin were used as the controls. As shown in the Fig. 3a, filtration with these uncoated control filters reduced the viable cell numbers by approximately one log. With filters coated with 0.9, 1.2, and 1.5 mg MWCNTs1 loading, the viable cell numbers were reduced by 1.93, 2.12, and 2.43 log after filtration, respectively. With filters coated with 0.9, 1.2, and 1.5 mg MWCNTs2 loading, the viable cell numbers were reduced by 2.69, 3.18, and 3.88 log after filtration, respectively. These results demonstrated that both types of MWCNT filters were effective on bacterial capture. While the MWCNTs2 filters were more effective than MWCNTs1 filters at each same loading level, in both cases, the higher MWCNTs loadings had a better bacteria capture efficiency. The bacterial capture efficiencies of these filters are comparable to those previously reported MWNTs, SWNTs, or SWNTs-MWNTs hybrid coated filters [38–40].

The high bacterial capture efficiency was attributed by the reduced pore sizes resulting from MWCNT coating on the membranes and the adsorption property of MWCNTs to bacterial cells. Various previously reported CNTs-coated filters/membranes have demonstrated the excellent potential of CNTs as bacterial capturing agents since they can spontaneously adsorb a significant amount of bacteria onto their surfaces [11]. However, it is also noted that besides MWCNTs’ properties, factors from the bacterial side, such as bacterium species, size, and shape, can also affect bacterium adsorption amount on CNTs. For instance, the smaller size *S. aureus* showed a five to ten times faster diffusion rate than *E. coli* and had about 100 times higher adsorption affinity with the CNT aggregates in water [41].

Figure 3b shows the log reductions in viable cell numbers in the filtrates after filtration with nisin-MWCNTs coated filters with various amounts of nisin deposit. As shown in Fig. 3b, nisin deposit on both types of MWCNT filters did not have an obvious influence on bacterial capture efficiency. However, on both types of MWCNTs-nisin filters, a clear trend was observed in which the bacteria capture efficiency was slightly reduced as nisin amount increased, possibly because the bacteria capture efficiency was negatively correlated with the hydrophobicity of filter surface. This phenomena was observed in previous studies reported by our group, where we observed that the surface hydrophobicity of MWCNT-nisin sheet was negatively correlated with *B. anthracis* spore attachment [27], and by others [42] who observed that hydrophilic uncharged surfaces had the greatest resistance to protein adsorption and cell attachment.

### Viability and Metabolic activities of bacteria captured on MWCNT-nisin filters

**Fig. 4:**
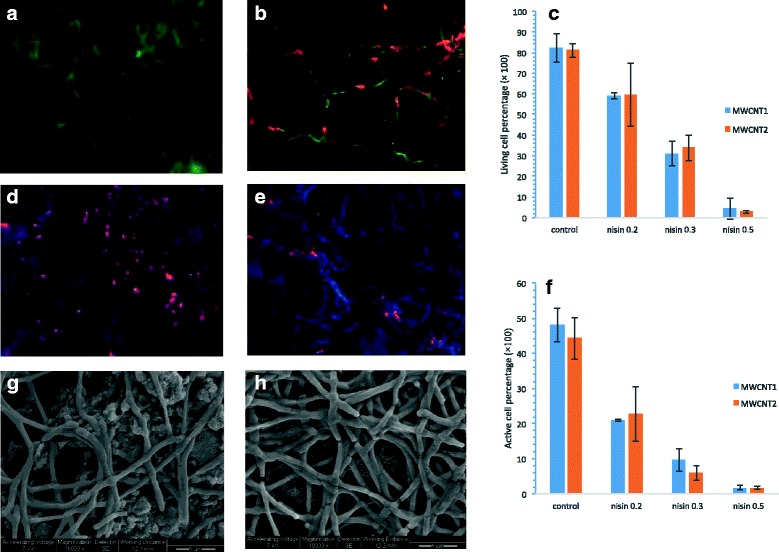
The viability and activity tests for *B. anthracis* cells on the MWCNT filters with or without nisin, as well as the SEM images of *B. anthracis* cells on MWCNTs-nisin filters. **a** A representative image of B. anthracis cells stained with bacterial Live/Dead kit on a non-nisin MWCNTs1 filter; (**b**) A representative cells image from the Live/Dead cell assay on a MWCNT1 filter with 0.3 mg nisin coating. **c** Live cell percentages on the filters deposited with different amounts of nisin calculated based on fluorescent images from the Live/Dead cell assay. **d** A representative image of *B. anthracis* cells with the CTC and DAPI staining on a MWCNT1 filter without nisin coating; (**e**) A representative cells image from the CTC and DAPI staining on a MWCNT1 filter with 0.3 mg nisin coating. **f** Metabolically active cell percentages on the filters deposited with different amounts of nisin calculated based on the fluorescent images from the CTC and DAPI staining test. **g** and (**h**) SEM images of *B. anthracis* cells that were trapped on the MWCNTs1 and MWCNTs2 coated filters, respectively

The functionalization with nisin on MWCNTs coated filters afforded the filters with bacterial inactivation function in addition to their bacterial capturing function. In order to examine the inactivation function of the resulting filters, the viability and metabolic activity of *B. anthracis* cells trapped on functionalized filters were tested. Figure 4a and b show representative fluorescent images of *B. anthracis* cells captured on a non-nisin MWCNTs1 filter and a nisin (0.3 mg)-MWCNTs1 filter stained with the Live/Dead BacLight Bacterial Viability kit. Live cells were stained in green and dead cells were stained in red. Figure 4c shows the quantitative analysis results of the images for the percentage of live cells on the filters. Without nisin, the percentage of living cell numbers was 82.18 and 81.09 % on MWCNTs1 and MWCNTs2 filters, respectively, indicating that MWCNTs coating only had a low inhibitory effect on captured cells. With nisin deposit, the living cell percentages were much lower than those on MWCNTs coated filters, and both types of filters showed an increased trend in the inactivation of cells with increasing nisin amount. Both types of filters showed very similar percentages of living cells on 0.2, 0.3, and 0.5 mg nisin deposited filters, with 59.04, 31.13, and 4.29 % on MWCNTs1-nisin filters, and 59.5, 33.80, and 2.81 % on MWCNTs2-nisin filters.

The results clearly demonstrated that functionalization with nisin effectively inactivated the captured bacteria cells on the MWCNT-nisin filters, causing a large amount of *B. anthracis* cell death. Based on previous studies, nisin inhibits the outgrowth of bacterial spores and the growth of vegetative Gram-positive bacteria through a mechanism of nisin binding to lipid II, which disrupts cell wall biosynthesis and facilitates pore formation [43].

The inactivation effect of nisin-MWCNTs filters on *B. anthracis* cells observed in this study is consistent with a number of previously reported studies on various nisin-incorporated films/surfaces. For example, studies have shown that nisin incorporated into polyethylene films or polylactic acid polymer films significantly inhibited/inactivated foodborne bacterial pathogens, reducing lactic acid bacterium numbers and *B. thermosphacta* numbers on beef after 1 h contact [25]. A nisin incorporated biodegradable film significantly inhibited the growth of *L. monocytogenes* in culture medium and in liquid egg white, and reduced viable *E. coli* O157:H7 cell numbers in orange juice by ~4 log in 72 h [26]. Nisin coating on a food packaging film was also shown to have inhibitory effects on *Micrococcus luteus* ATCC 10240 in tryptone soya broth and the bacterial flora in milk [44]. Nisin-mixed polylactic acid polymer coating on glass jars rapidly reduced viable *Listeria monocytogenes* in inoculated liquid egg white and skim milk to undetectable levels after 1 day. Our previous studies found that nisin adsorption on the MWCNT sheets reduced the germination of attached *B. anthracis* spores by 3.5 fold, and further inhibited the subsequent biofilm formation by 94.6 % compared to that on the MWCNT sheet without nisin [27]. The results reported in this study have proven that nisin could be incorporated with MWCNT-coated membranes/filters to provide desired antibacterial function. The results have also validated this developed approach in providing dual functions for capturing and inactivating pathogens in one single filtration step.

Further tests on the metabolic activities of the *B. anthracis* cells captured on the MWCNT filters and the MWCNT-nisin filters were performed by 5-cyano-2,3-ditolyl-tetrazolium chloride (CTC) staining. CTC is one of the most widely used tetrazolium salts for the detection of dehydrogenase-dependent metabolic activities in microbial cells. CTC is converted to fluorescent CTC-formazan precipitates when reduced through biological redox reactions, for example, respiratory electron transport [45]. The CTC staining method is responsible for the bulk of microbial redox reactions or metabolism in which multi-enzymes are involved [45], and is different in principle from the Live/Dead viability assay. Figure 4d and e show representative fluorescent images of *B. anthracis* cells on a non-nisin MWCNTs1 filter and a nisin (0.3 mg) deposited MWCNTs filter stained with CTC method. Metabolically active cells were stained in red and inactive cells were stained in blue. Figure 4f shows the quantitative percentages of metabolically active cells in the total number of live cells. The CTC staining results indicated that among the live cells on the filters, only a certain percentage of cells were metabolically active on each type of filter. On non-nisin deposited MWCNTs1 and MWCNTs2 filters, 58.44 and 54.67 % of live *B. anthracis* cells were metabolically active, respectively. On the MWCNT-nisin filters, 35.63 and 38.17 % of live cells showed metabolic activity on 0.2 mg nisin deposited MWCNT1 and MWCNT2 filters, respectively. Previous research also found that there were more live *E. coli* cells than metabolically active cells on the SWCNTs filters [30]. Similar to the trend of living cell percentages, the percentage of metabolically active cells decreased with increasing nisin amount deposited on the filters, with 21.03 % on 0.2 mg nisin deposited MWCNTs1 filters and 1.78 % on 0.5 mg nisin deposited filters. The MWCNTs2-nisin filters resulted in similar percentages of metabolically active cells with 1.7 % on 0.5 mg nisin deposited filters. These results indicated that nisin decreased metabolic activities in *B. anthracis* cells which also agreed with other studies that observed the immediate inhibitory effect of nisin Z on the respiratory activities of *Listeria monocytogenes* cells [46].

Figure 4g and h show the representative SEM images of *B. anthracis* cells trapped on the MWCNTs-nisin filters. The trapped cells still kept their chain-forming morphology. No cell morphology changes were observed on the MWCNT filters and the MWCNT-nisin filters, which is unlike the obvious cell membrane damage observed on bacterial cells trapped on SWCNT or MWCNT filters previously reported. This is possibly because the bacterial species are different, and the contact time may make a difference too. It is reported that the lytic-bactericidal lantibiotic nisin can cause multiple aberrations, including leakage of cytoplasmic contents, reduction of cell width, acceleration of cell division, minicell formation, abnormal morphogenesis of bacterial cells and eventual cell death [47], and increased bacterial elongation time and a changed bacteria morphology near the division site [48]. Most of these changes were observed during nisin treatment to bacterial cells in aqueous solutions. In the case of bacterial cells trapped on the MWCNT-nisin filters, only a limited area of a cell surface was contacted with nisin molecules, and such contacts/interactions were sufficient to inhibit metabolic activity and even inactivate cells, but may have not resulted in directly damage of the cell membranes.

## Conclusions

This study demonstrated that MWCNTs-nisin coated filters possess dual functions in capturing and inactivating bacterial pathogens. The high adsorptivity of MWCNTs assisted to adsorb and retain the natural bactericidal nisin on the filters and also enhanced the capturing of bacterial cells on the coated filters. Based on the tests in this study, with 1.5 mg MWCNTs loading, the MWCNT1filters and MWCNT2 coated filters captured 2.43 and 3.88 log of *B. anthracis* cells from aqueous solutions containing a total of 10^6^ CFU/mL cells, respectively. The difference in MWCNTs properties may result in a difference in absorption of nisin and capture of bacterial cells, but it is believed to be adjustable to optimal conditions accordingly. Nisin deposit at the amount of 0.5 mg on the surfaces of MWCNT filters significantly reduced the viability of captured *B. anthracis* cells by 95.71–97.19 %, and inhibited the metabolic activities of the retained cells by approximately 98.3 % on the MWCNTs-nisin filters. The results demonstrated that the MWCNT-nisin filters achieved bacteria capture and inhibition in one single filtration step, which shows potential in improving water quality by removing undesired microorganisms from water sources and inhibiting the retained Gram positive bacteria activities.

## Methods

### MWCNT-nisin filter preparation

MWCNTs at two different sizes (denoted as MWCNTs1 and MWCNTs2) were purchased from NanoIntegris Inc. (Skokie, IL, USA). Both types of MWCNTs had the purity of >95 wt% and the length of 10–30 μm. MWCNTs1 had an outer diameter of < 8 nm, an inner diameter of 2–5 nm, and a specific surface area of 500 m^2^/g. MWCNTs2 had an outer diameter of 10–20 nm, an inner diameter of 3–5 nm, and a specific surface area of 233 m^2^/g.

Isopore polycarbonate hydrophilic membrane filters (catalog: TMTP membranes) with a diameter of 25 mm and pore size of 5 μm were purchased from EMD Millipore (Billerica, MA). For coating the filters with MWCNTs, the as-received MWCNTs1 and MWCNTs2 were suspended in dimethylsulfoxide (DMSO) at the concentration of 3 mg/mL. The suspensions were sonicated for 10 min and then deposited onto TMTP membranes with the desired MWCNTs loadings. The membranes were air dried, and washed with ethanol by filtering 5 mL of 100 % ethanol at a flow rate of 0.5 mL/min using a syringe pump to remove residual DMSO, and then by filtering 10 mL of deionized (DI) water at a flow rate of 1.5 mL/min to remove ethanol.

Nisin was purchased from Fisher Scientific International, Inc (Pittsburgh, PA, USA). Nisin solutions were prepared by dissolving the desired amount of nisin in 0.01 mol/L NaH_2_PO_4_ solution and then adding sufficient 0.01 mol/L Na_2_HPO_4_ solution to reach the final pH value of 7.0. For preparation of nisin-MWCNTs filters, the above MWCNTs coated filters with 1.2 mg MWCNTs1 or MWCNTs2 loading were used. 200 μL of nisin solution at various concentrations were loaded onto the filters to achieve final loadings of nisin at 0.2, 0.3, or 0.5 mg on the filters. The nisin-MWCNTs filters were then air dried for 30 min at room temperature and then washed with 3 mL DI H_2_O by filtration to remove un-adsorbed nisin. The resulting filters were air dried for another 20 min and used immediately in experiments.

### Characterization of MWCNTs coated filters and nisin-MWCNTs filters

The surface morphology and the thickness of the coating of MWCNTs1 and MWCNT2 on the filters were determined by scanning electron microscopy (SEM). All SEM images were taken using the FEI XL30 microscope (Netherlands) at the Shared Materials and Instrumentations Facility (SMIF) at Duke University. For filters with bacterial cells, the cells were first fixed in bacterial fixative (4 % formaldehyde and 2 % glutaraldehyde in 1x PBS) for 6 h at room temperature. After washing with DI water, the samples were air-dried. Filters without bacterial cells did not need the fixing step. Before SEM imaging, all filters were coated with gold using Denton Vacuum Desk IV (Czech Republic) as previously described [49].

The hydrophilic property of the MWCNTs coated filters and nisin-MWCNTs filters were examined by measuring the contact angles of DI water on the filters using the water sessile drop method on a goniometer (Ramé-hart instrument co., Succasunna, NJ, USA). The mean contact angles were calculated based on a drop of DI H_2_O on the surface with at least five replicates. Statistical analysis was performed using the SAS System 9.1 (SAS Institute Inc., Cary, NC, USA).

### Bacteria capture and cell viability tests

*B. anthracis* Sterne 34 F2 cells (Colorado Serum Company, Denver, CO) were freshly grown in Nutrient broth at 37 °C overnight. The cell culture was centrifuged and the cell pellet was washed three times with DI water and then resuspended in sterilized tap water. The cell number in the suspension was determined by the traditional plating method using Luria-Bertani (LB) agar plates. To evaluate the efficiencies of MWCNT coated filters with or without nisin, 2 mL of cell suspension was filtered through the freshly prepared filters at a flow rate of 0.5 mL/min using a syringe pump, the filtrate was collected and the cell number in the filtrate was determined by the plating method on LB agar plates. The viable cell number was calculated in colony forming units per milliliter (CFU/mL). The reduction of viable cell number in the filtrate in comparison to that before filtration was the parameter for evaluating the performance of coated filters for capturing of bacterial cells from water. All tests were carried out in triplicates.

The viability and metabolic activity of the cells trapped on the filters were examined. After filtration, the filters were sat at room temperature in the dark for 30 min before cell viability and metabolic activity tests. For the viability test, the Live/Dead BacLight Bacterial Viability kit (Invitrogen, USA) was used to stain the cells on the filters according to the protocol from the manufacturer. The fluorescent images were taken on a fluorescent microscope (Nikon ECLIPSE E600FN, Japan) with a Coolsnap HQ camera (Roper Scientific Inc., −Photometric, Tucson, AZ). The percent of inactivated cells was determined from at least 8 representative images of each sample and calculated from the ratio of the cells stained with propidium iodide (PI, red) to the cells stained with SYTO 9 (green) plus PI stained cells using the software ImageJ (National Institutes of Health, Bethesda, MD) [50]. For the metabolic activity tests, 1 mL of M63 medium solution was added to the cells trapped on the filters and incubated for 30 min. The cells were then stained with 6 mM 5-cyano-2,3-ditolyl-tetrazolium chloride (CTC, Sigma-Aldrich, U.S.A.) at 37 °C for 1 h, followed by DAPI staining for 5 min according to a previous study [30]. The percentage of metabolically active cells was calculated by dividing the cells stained with CTC (red) by the total number of cells stained with DAPI (blue) plus CTC stained cells [30, 51].

## Abbreviations

CFU/mL: Colony forming units per milliliter
CNTs: Carbon nanotubes
CTC: 5-cyano-2,3-ditolyl-tetrazolium chloride
DI: Deionized water
LB: Luria-Bertani agar
DMSO: Dimethylsulfoxide
MWCNTs: Multi-walled carbon nanotubes
PI: Propidium iodide
SEM: Scanning electron microscopy
SWCNTs: Single-walled carbon nanotubes
